# Hybrid diffusion imaging reveals altered white matter tract integrity and associations with symptoms and cognitive dysfunction in chronic traumatic brain injury

**DOI:** 10.1016/j.nicl.2021.102681

**Published:** 2021-04-21

**Authors:** Jennifer Muller, Devon Middleton, Mahdi Alizadeh, George Zabrecky, Nancy Wintering, Anthony J. Bazzan, Ji Lang, Chengyuan Wu, Daniel A. Monti, Qianhong Wu, Andrew B. Newberg, Feroze B. Mohamed

**Affiliations:** a Jefferson Integrated Magnetic Resonance Imaging Center, Department of Radiology, Thomas Jefferson University, Philadelphia, PA, USA; b Department of Integrative Medicine and Nutritional Sciences, Marcus Institute of Integrative Health, Thomas Jefferson University, Philadelphia, PA, USA; c Cellular Biomechanics and Sports Science, Department of Mechanical Engineering, Villanova University, Villanova, PA, USA; d Department of Neurological Surgery, Vickie and Jack Farber Institute for Neuroscience, Thomas Jefferson University, Philadelphia, PA, USA

**Keywords:** Traumatic brain injury, Hybrid diffusion imaging, Post-concussion syndrome, Diffusion tensor imaging, Neurite orientation dispersion and density imaging, Multi-band imaging

## Abstract

The detection and association of in vivo biomarkers in white matter (WM) pathology after acute and chronic mild traumatic brain injury (mTBI) are needed to improve care and develop therapies. In this study, we used the diffusion MRI method of hybrid diffusion imaging (HYDI) to detect white matter alterations in patients with chronic TBI (cTBI). 40 patients with cTBI presenting symptoms at least three months post injury, and 17 healthy controls underwent magnetic resonance HYDI. cTBI patients were assessed with a battery of neuropsychological tests. A voxel-wise statistical analysis within the white matter skeleton was performed to study between group differences in the diffusion models. In addition, a partial correlation analysis controlling for age, sex, and time after injury was performed within the cTBI cohort, to test for associations between diffusion metrics and clinical outcomes. The advanced diffusion modeling technique of neurite orientation dispersion and density imaging (NODDI) showed large clusters of between-group differences resulting in lower values in the cTBI across the brain, where the single compartment diffusion tensor model failed to show any significant results. However, the diffusion tensor model appeared to be just as sensitive in detecting self-reported symptoms in the cTBI population using a within-group correlation. To the best of our knowledge this study provides the first application of HYDI in evaluation of cTBI using combined DTI and NODDI, significantly enhancing our understanding of the effects of concussion on white matter microstructure and emphasizing the utility of full characterization of complex diffusion to diagnose, monitor, and treat brain injury.

## Highlights

• Hybrid Diffusion Imaging (HYDI) detects white matter associations in patients with cTBI.
• The advanced diffusion model NODDI was more sensitive in detecting between-group differences than classic DTI.
• DTI appeared to be just as sensitive as NODDI for detecting white matter correlations with self-reported symptoms.
• This study highlights the advantages of acquiring both DTI and NODDI to fully characterize white matter microstructure in cTBI.

## 1 Introduction

Traumatic brain injury (TBI) is a significant public health problem which occurs as a result of multiple incidents, including vehicle accidents, falls, athletic collisions, blast-related trauma, and abuse or assault. (Asken et al., 2018) Symptoms of TBI include a range of short- and long-term adverse clinical outcomes, including cognitive impairments or emotional dysregulation, resulting from traumatic axonal injury. (Kraus et al., 2007) Despite the increasing evidence that mTBI causes axonal shearing of white matter (WM) microstructure, the lack of reliable and objective tools to measure this pathology is a barrier to clinical translation. (Levin and Diaz-Arrastia, 2015, Radhakrishnan et al., 2016) As a result, it is commonly assumed that subjects with mTBI will return to premorbid levels of functioning shortly after the traumatic event, which often results in insufficient follow-up care. (Yamagata et al., 2020).

Diffusion tensor imaging (DTI) is a non-invasive magnetic resonance imaging (MRI) technique for assessing WM microstructure in vivo, and has revealed diffuse axonal injury in TBI subjects in the absence of injury signs on conventional MRI. (Maruta et al., 2016) DTI studies of mTBI have shown microstructural disruption to be associated with neurocognitive and behavioral deficits after mild and chronic TBI. (Yamagata et al., 2020, Wu et al., 2018, Wallace et al., 2018) However, traditional DTI metrics represent basic statistical descriptions of diffusion that may not directly correspond to biophysically meaningful parameters of the underlying tissue. (Palacios et al., 2018) Moreover, DTI assumes Gaussian diffusion within a single microstructural compartment, and may be insensitive to the complexity of WM structure which requires a non-Gaussian model with multiple compartments. (Jones and Cercignani, 2010) Although WM differences in mTBI have been delineated using DTI, the magnitude, direction, locations, and time span of these changes have been inconsistent among studies. (Wu et al., 2018, Inglese et al., 2005) For example, several papers report reduced WM fractional anisotropy (FA) in mTBI, while others reporting elevations or no changes in FA. (Eierud et al., 2014) This can be attributed to a number of reasons, including the inherent dynamic nature of microstructural WM alterations after mTBI, heterogenous population phenotypes, and small sample sizes.

Due to the complexity of chronic and acute TBI, the combination of higher-order biophysical measurements based on diffusion MRI has the potential for better characterizing the underlying microarchitectural changes in brain tissue. (Palacios et al., 2020a, Palacios et al., 2020b) A more advanced multicompartment diffusion model known as neurite orientation dispersion and density imaging (NODDI) utilizes high-performance magnetic field gradients to probe more complex non-Gaussian WM diffusion, and measures the properties of three microstructural environments: intracellular, extracellular, and free water. (Zhang et al., 2012) An increasing number of recent studies have applied NODDI to examine white matter changes following mTBI. (Wu et al., 2018, Palacios et al., 2018, Mayer et al., 2010, Churchill et al., 2017) Findings in these papers reveal that NODDI metrics may be more sensitive and likely influenced by different factors than DTI metrics, providing more sensitive and useful diagnostic information. (Gazdzinski et al., 2020) To translate research findings into clinical practice, replication and generalization of these diffusion sequences are essential, (Lerma-Usabiaga et al., December 2018) with neuroimaging findings reproducible in an independent dataset acquired under real-world conditions.

Hybrid diffusion imaging (HYDI) is a comprehensive diffusion sequence (Alexander et al., 2006) comprising multiple diffusion-weighting shells which offers diffusion compartments sensitive to different diffusivities and multiple diffusion-weighting directions in each shell to capture the directionalities of each compartment. In HYDI, multiple sampling spheres in q-space offer data needed for a range of diffusion reconstruction methods- such as DTI, NODDI, q-ball imaging (QBI), and diffusion spectrum imaging (DSI). (Wu et al., 2018) Furthermore, HYDI utilizes lower b-value shells with high angular contrast-to-noise ratios, offering a better characterization of complex tissue organization. (Daianu et al., 2015) Moreover, unlike other studies of both DTI and NODDI, the HYDI sequence is advantageous as multiple models can be fit using a single acquisition, decreasing total imaging time especially when combined with simultaneous multi slice acquisition, making it feasible in a clinical setting. Additionally, comparisons between the modeling techniques may be more accurate, as all models are being created from a single acquisition reducing confounds of signal noise and motion. Therefore, the application of this technique represents a novel contribution over other available work on cTBI utilizing multi-shell diffusion imaging. In this study, we used a five-shell HYDI to sample full q-space diffusion signals. The HYDI data was used to extract six diffusion metrics computed from the DTI and NODDI model, in a large well-phenotyped cohort of cTBI subjects. We aim to compare and evaluate the extent at which in both NODDI and DTI measurements correlate to self-reported symptoms in mTBI within a chronic population, to further validate diffusion biomarkers and explore the prognostic significance of advanced imaging techniques.

## 2 Methods

### 2.1 Participants

A total of 40 subjects including 12 males (age: 46 ± 19.5 years) and 28 females (age: 49 ± 15.8 years) experiencing chronic symptoms caused by a mild traumatic brain injury were included in this study. mTBI was defined by the Mayo Classification System for Traumatic Brain Injury Severity, in which an injury was classified as mild if loss of consciousness of momentary was <30 min, amnesia for <24 h, with no positive MRI findings. (Malec et al., 2007) 14 of the 40 cTBI subjects had sustained a single concussion, with 26 of subjects having experienced multiple concussions. We compared the cTBI subjects to 17 healthy control subjects including 10 males (age: 32 ± 8.8 years) and 7 females (31 ± 10.5 years). Written informed consent, approved by the Institutional Review Board, was obtained from all subjects and the study was registered on clinicaltrials.gov with the following identifier: NCT03241732. Subjects were recruited from the local community by self-referral and from local neurology offices and were excluded if they had a history of other neurological disorders, significant medical illness, a current substance-use disorder, or current *Diagnostic and Statistical Manual of Mental Disorders, 4th Edition* (DSM-IV) Axis I psychiatric illness. Subjects had to report a history of one or more prior TBIs with symptoms that lasted at least 3 months apart from the last concussion. All subjects had to meet criteria for mild traumatic brain injury including: loss of consciousness < 30 min, no significant amnesia, and no structural injury to the brain such as hematoma, contusion, dura penetration, or brain stem injury. Symptoms had to result after the TBI and could include headache, hypersensitivity to auditory or visual stimuli, balance problems, cognitive problems, or emotional problems (i.e. depression or anxiety).

For the control group, individuals were excluded if they had a history of previous TBI, a history of other neurological disorders, significant systemic medical illness, a current substance-use disorder, and current *Diagnostic and Statistical Manual of Mental Disorders, 4th Edition* (DSM-IV) Axis I psychiatric illness.

### 2.2 Neuropsychological assessment

Clinical assessment of TBI subjects experiencing chronic symptoms included a battery of self-reported measures including the State-Trait Anxiety Inventory, Beck Depression Inventory, Profile of Mood Scale, Rivermead Post Concussion Symptoms Questionnaire (RPQ-3 and RPQ-13), the Epworth Sleepiness Scale, and two cognitive tests – the forward and backward digit span, and the Trails A and B test. Clinical assessments were performed on the same day of the imaging study. Details of the neuropsychological measures and patient demographics are listed in Table 1. The clinical and neuropsychological measures were correlated with the diffusion metrics across the whole-brain white matter skeleton.

**Table 1:** Subject demographic and neuropsychological measures, averages and standard deviations reported across control and mTBI subjects.

	Control	Mild TBI
**Demographics**	*n = 17*	*n = 40*
Age (year)(std)	33.2 (10.9)	48.0 (16.8)
Sex (M:F)	10:7	12:28
Injury-to-imaging interval (months)(std)	–	73.0 (117.8)
Single concussion vs. multiple (single:multiple)	–	14:26
**Neuropsychological Measures**		(mean ± std)
State Trait Anxiety Inventory		
State Anxiety		44.3 ± 14.1
Trait Anxiety		44.7 ± 12.5
Back Depression Inventory		17.1 ± 11.0
Profile of Moods Scale		
Tension		12.3 ± 8.0
Depression		12.3 ± 13.6
Anger		7.9 ± 6.6
Vigor		11.0 ± 6.1
Fatigue		12.4 ± 7.2
Confusion		11.3 ± 5.6
Mayo-Portland Adaptability Inventory-4		
Ability Index		15.6 ± 7.8
Adjustment Index		17.5 ± 9.0
Participation Index		8.4 ± 5.5
Total		35.4 ± 16.3
Rivermead		
RPQ-3		5.1 ± 2.5
RPQ-13		26.9 ± 10.5
Digit Span		
Forward		10.5 ± 2.1
Backward		7.2 ± 2.3
Epworth Sleepiness Scale		7.7 ± 5.0
Trail Making (seconds to complete)		
A		28.0 ± 10.8
B		65.5 ± 21.5

### 2.3 Imaging protocol

In vivo brain data with HYDI was obtained on 17 healthy volunteers and 40 chronic traumatic brain injury subjects using a 3T Siemens Biograph MR PET-MR scanner with a 32-channel head coil. For segmentation and registration of white matter atlas structures, and to check whether or not any conventional positive radiological findings of brain injury could be detected, an anatomical T1-image was obtained for all cTBI and healthy control subjects. MRI parameters for the anatomical T1-weighted sequence were as follows: repetition time = 1.6 s, echo time = 2.46 ms, field of view = 250 × 250 mm, matrix = 512 × 512, voxel size = 0.49 × 0.49 mm (Kraus et al., 2007), 176 slices with slice thickness = 1 mm. The simultaneous multi-slice (SMS) HYDI pulse sequence was a single-shot, spin-echo, echo-planar imaging (SS-SE-EPI) pulse sequence with diffusion gradient pulses. The minimum b-value was 0 sec/mm (Kraus et al., 2007) with five concentric diffusion-weighting shells (b-values = 250, 1000, 2000, 3250, 4000 sec/mm^2^). A total of 144 diffusion-weighting gradient directions (6, 21, 24, 30, and 61 in each shell) were encoded. MRI parameters for the HYDI sequence were as follows: repetition time = 3.17 sec, echo time = 120 ms, field of view = 240 × 240 mm, matrix = 96 × 96, voxel size = 2.5 × 2.5 mm^2^, 63 slices with slice thickness = 2.5 mm, simultaneous multi-slice factor = 2, and total scan time of 8 min. Diffusion parameters included a maximum b-value of 4000 sec/mm^2^.

### 2.4 Imaging processing

**Fig. 1:**
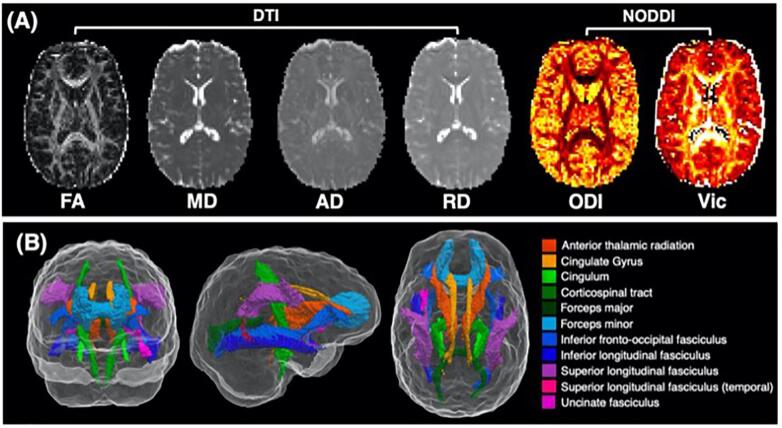
Maps of diffusion metrics in a single representative subject (A). Diffusion tensor imaging (DTI) metrics include fractional anisotropy (FA), axial diffusivity (AD), radial diffusivity (RD), and mean diffusivity (MD). Neurite orientation dispersion (ODI) and axonal density (V_ic_). White matter masks registered to subject space from the JHU atlas (B).

Image processing included an initial pre-processing of the raw DICOM data, and a computation of diffusion metrics. First, the susceptibility induced distortion was estimated and corrected for using the topup tool provided in the eddy current correction method of the FMRIB Software Library (FSL). (Jenkinson et al., 2012) The topup output was fed into the eddy tool by aligning all volumes to the b0 image. DTI parameter maps were calculated using the FSL Diffusion Toolbox DTIFIT. Additionally a MATLAB based toolbox (https://www.nitrc.org/projects/noddi_toolbox) was used to compute higher order diffusion metrics from the NODDI component of the analysis were analyzed including neurite density, also known as intra-cellular volume fraction (V_ic_) and the orientation dispersion index (ODI). The resulting FA, axial diffusivity (AD), radial diffusivity (RD), mean diffusivity (MD), V_ic_, and ODI maps in a single representative patient are shown in Fig. 1**A**.

## 3 Statistics

### 3.1 Tract-based spatial statistics WM voxelwise analysis

After calculation of the six diffusion maps, a voxelwise statistical analysis of the data was performed using tract-based spatial statistics (TBSS). Diffusion maps were aligned to a common template in MNI152 (Montreal Neurological Institute) standard space, using a nonlinear registration algorithm FNIRT. Next, a mean image was created from all the images for all subjects serial scans in standard space and thinned to generate a mean WM skeleton representative of all tracts common to the entire group of scans. The aligned volumes were then projected onto the skeleton by filling the skeleton with values from the nearest relevant tract center. Output images and the 0.2 thresholded skeleton maps were visually inspected for accuracy.

We compared between subjects using a voxelwise general linear model (GLM) analysis with permutation testing to correct for multiple comparisons using familywise error corrected at p < 0.05, and threshold-free cluster enhancement. A paired *t*-test was used to compare differences among DTI and NODDI measures between TBI and control subjects. Age, sex, and time interval between injury and imaging date were included as nuisance covariates. The significance maps were then broken into twenty regions using masks obtained from the Johns Hopkins University white-matter tractography atlas mapped onto the standard MNI152 space.

### 3.2 Partial correlation analysis

To examine the correlation of diffusion parameters indicating white matter microstructure abnormalities with cognitive function in TBI subjects, Spearman rank partial correlation coefficients between all diffusion metrics and each of the twenty neuropsychological tests was generated for all subjects within the TBI cohort (n = 40). Partial correlations were generated between regional white matter diffusion metrics, with performance in neuropsychological testing, controlling for age, sex, and the time interval between injury and imaging date. A significance value was determined using a Student’s *t* distribution, with the linear correlation being considered significant if p < 0.05.

## 4 Results

### 4.1 Between-group differences in the diffusion metrics

**Fig. 2:**
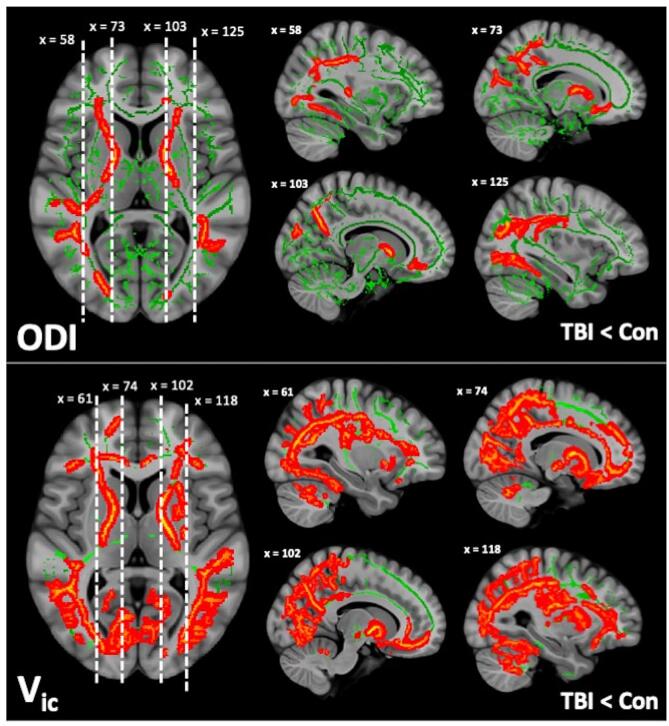
TBSS maps of significant differences of ODI (A) and V_ic_ (B) between TBI and healthy controls. Red voxels indicate regions with significantly lower values in TBI versus controls (p < 0.05), green voxels indicate no significant differences. (For interpretation of the references to color in this figure legend, the reader is referred to the web version of this article.)

Averaged maps of FA, AD, RD, MD, ODI, and V_ic_ across all subjects were tested by TBSS. Among the six different diffusion metrics tested by TBSS, only NODDI metrics (ODI And V_ic_) differed significantly between groups (Fig. 2), with individuals with cTBI having both lower ODI and V_ic_ than the normal control group. No clusters were found to be significantly higher in the TBI population than the control group for ODI and V_ic_. Approximately 11.20% of total skeletonized voxels for ODI and approximately 15.73% of V_ic_ voxels were found to be significantly lower across the whole brain in the TBI population when compared to the normal control group. These significant results (corrected *p* < 0.05) were detected in 15 of the 20 white matter tracts of the JHU atlas, and the affected tracts were located predominately in the forceps minor as well as the superior and inferior longitudinal fasciculus (Fig. 2).

**Fig. 3:**
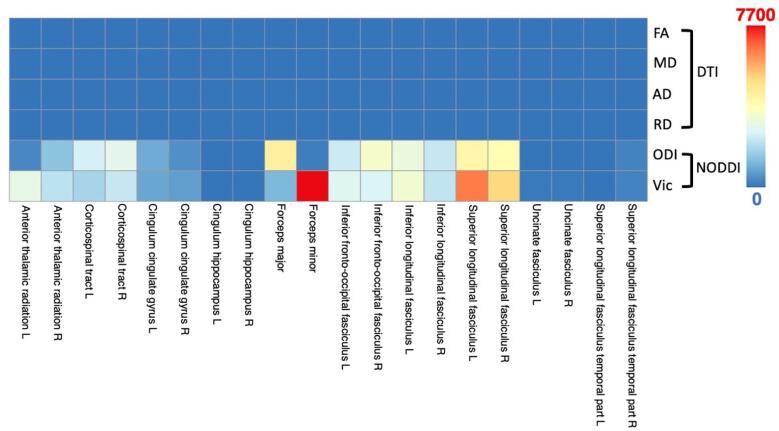
Results of the TBSS analysis using permutation testing for group comparisons. Listed are the brain locations of significant voxels, the names of the major fiber tract, the color representing cluster size. Significance was determined at p < 0.05.

Significantly different voxels were broken into clusters via the white matter atlas with *p* values ranging from 0.049 to 0.017. Affected white matter tracts were located predominantly in the superior and inferior longitudinal fasciculus, as well as the forceps major and minor and along the corticospinal tracts. The detection of statistically significant between-group differences, and corresponding cluster size is shown in Fig. 3.

### 4.2 Relationship of diffusion metrics to cognitive function: Within-group correlations

**Fig. 4:**
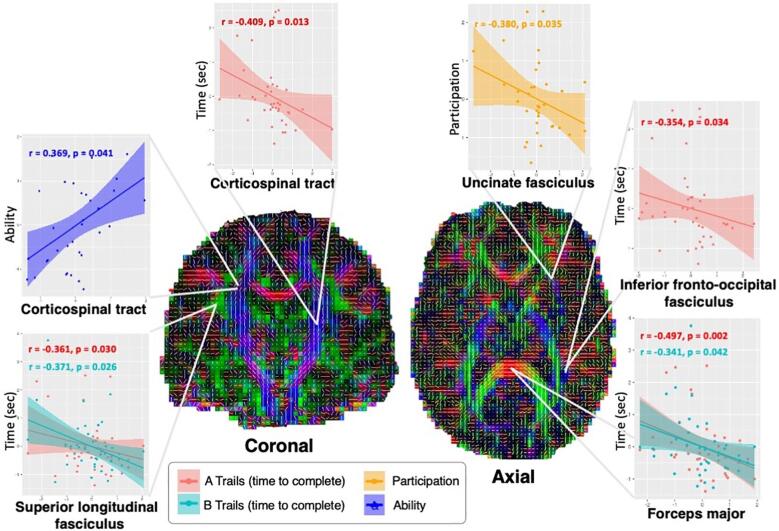
Results of post-hoc regression analysis for the six FA-neuropsychological pairs that showed significant correlations. Regional residual FA values are displayed on the x-axis, displaying the effects of controlling for age, sex, and time after injury. Neuropsychological correlations are displayed on the y-axis. Complete results for correlations between diffusion metrics and neuropsychological outcomes are displayed in Fig. 5.

Within the cTBI cohort, diffusion metrics FA, MD, AD, RD, ODI, and V_ic_ had a wide spread of white matter regions (14 out of the 20 regions defined by the JHU atlas) that were either positively or negatively correlated with neuropsychological outcomes (p < 0.05). The results of the correlation analysis for within-group correlations between FA and time to complete Trail Making (A and B) is shown in Fig. 4. The Trail Making Test a neuropsychological test of visual attention and task switching, sensitive to detecting cognitive impairment, and is a measure of executive function. (Arnett and Labovitz, 1995) An increased time to complete Trail Making (A or B) indicates a decrease in performance.

FA, MD, AD, and RD, correlated either positively or negatively with measures in mood, memory, and executive function depending on region. In general, as FA increased, performance on tasks increased (decreased time to complete trails, as seen in the negative correlation in Fig. 4). MD, AD, and RD had the opposite correlation with trail-making, with decreased performance correlated with increased measures. ODI had no significant correlation with trail-making, whereas V_ic_ had a similar relationship as FA, having a positive correlation with increased axonal density and task performance.

**Fig. 5:**
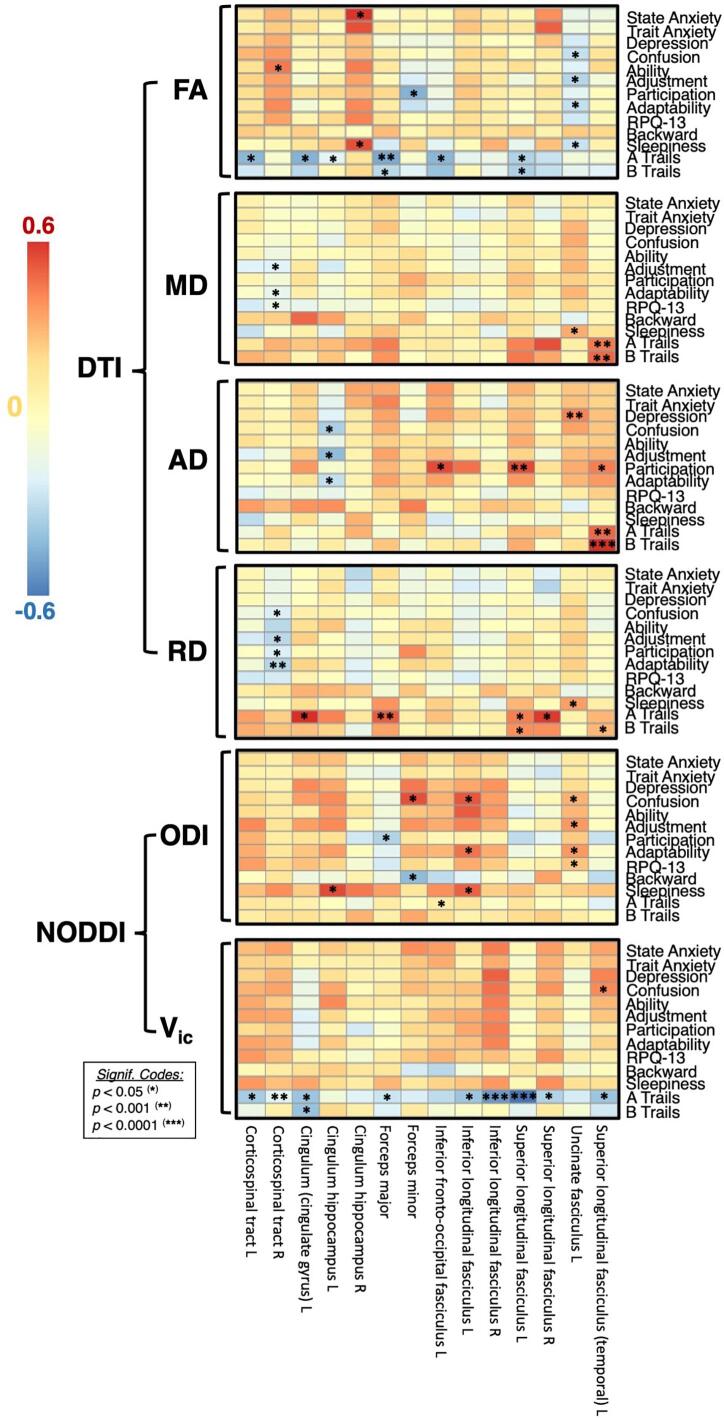
Scatter plot of significant correlations between DTI and NODDI metrics and neuropsychological tests (State Anxiety, Trait Anxiety, Depression, Confusion, Ability, Adjustment, Participation, Adaptability, RPQ.13, Backwards, Sleepiness, A Trails, B Trails) for all TBI subjects. For trail making (A and B) the time taken to complete the task is indicated on the y-axis, therefore a negative correlation would indicate increasing DTI or NODDI metrics correlated with an increase in performance. The horizontal axis of the matrix denotes the JHU white matter label, with the vertical axis representing the corresponding relationship with neuropsychological measures. The color and intensity denotes the strength of the correlation coefficient with corresponding p-values as described in Table 2. Red color indicating significantly positive correlations, blue indicating significant negative correlations. (For interpretation of the references to color in this figure legend, the reader is referred to the web version of this article.)

For all of the 76 significant diffusion-neuropsychological pairs, the extent of the correlation expressed as the correlation coefficient is summarized as a color-coded matrix in Fig. 5. V_ic_ correlated positively primarily with measures of executive function (trail making). ODI did not have correlations with task performance, however significant correlations were found with 7 different neuropsychological tests of mood and memory. For all measures, the atlas white matter region in which the measure was taken resulted in either a positive or negative correlation. The most sensitive measures for correlation with neuropsychological outcomes were FA and AD. FA had a positive correlation with performance in trail making (negative correlation with time to complete), similar to V_ic_. All other diffusion metrics including MD, AD, RD, and ODI were negatively correlated with performances in trail making.

## 5 Discussion

Here we demonstrate the advantages of modeling higher-order diffusion sequences using an advanced multi-shell HYDI sequence for detecting white matter injury in cTBI. The main findings of this study are (i) HYDI can be used to delineate WM alterations in cTBI using both NODDI and DTI using a single image acquisition (ii) NODDI is sensitive to white matter pathology in the posterior periventricular regions following cTBI, and can detect differences in voxels not detected by conventional DTI and (iii) both DTI and NODDI metrics are significant in partial correlations with neuropsychological outcomes, after controlling for age, sex, and time after injury.

Traumatic brain injury involves multiple different time-varying pathophysiological effects, including diffuse axonal injury, diffuse microvascular injury, and neuroinflammation, which can lead to neurologic dysfunction. (Bigler, 2015) Due to this complexity, the relationship between neurobiological response and the course of clinical recovery remains incompletely understood. (Churchill et al., 2019) Combining different biophysical measurements has potential for better characterization of the underlying microstructural changes in WM. (Cercignani and Bouyagoub, May 2017)

Using TBSS, we found that mild TBI subjects in the chronic phase showed significantly lower NODDI values in cTBI versus controls, mainly in the posterior and periventricular regions (Fig. 2). Tracts with low ODI values largely overlapped with tracts that exhibited low V_ic_ values, in cTBI subjects. In contrast, there were no group differences in DTI values including FA, AD, RD and MD (Fig. 3). This suggests that NODDI is a more sensitive biomarker for detecting longer-term alterations marked by declining neurite density in predominantly posterior WM in cTBI, than DTI. Our findings agree well with previous literature finding that NODDI parameters are more sensitive imaging biomarkers than traditional DTI for detecting the subtle yet complex underlying WM microstructural pathology after mTBI. (Wu et al., 2018, Churchill et al., 2019, Palacios et al., 2020a, Palacios et al., 2020b) The observed effects may be driven by multiple physiological responses to concussion, including glial-mediated edema which may be intracellular or vasogenic, and can lead to an increase in extra-neurite water volume. (Churchill et al., 2017) Our results showed lower ODI and V_ic_ specifically within the posterior periventricular regions, lesioning of which has been described to be disruptive to the overall integrative of the whole-brain WM network, and may be responsible for the long-term symptomatic cognitive, and behavioral outcomes seen after mTBI. (Xiao et al., 2016;10(OCT):1–12) This may be due to the disproportionately high number of structural connectome links between gray matter areas, forming a chain of network connectivity in the brain, as well as the presence of ventricles leading to strain concentration in the periventricular region, (Zhou et al., 2020) and may explain why subjects with lower ODI and axonal density may be experiencing cognitive deficits months to years prior to sustaining mTBI.

The inability of FA, MD, AD and RD to detect WM abnormalities highlights the discrepancies of DTI in the literature as a reliable biomarker for TBI. This pathological interpretation has been supported by mouse models, which have the advantage of using histological methods to investigate cellular processes that contribute to MRI measures. A study by Gadzinksi et al., found that ODI was more sensitive than FA in detecting damage from closed-skull impact in mice, and correlated with cellular changes identified by histological staining. They concluded that differences in ODI were associated with histological measures of astrogliosis, neuroinflammation and axonal degeneration, and persisted beyond behavioral impairment. Researchers concluded that in the context of a closed-skull impact animal model, NODDI is more sensitive to white matter pathology following mTBI and provides information not obtained from conventional DTI. (Gazdzinski et al., 2020) Our study translates these findings into a real-world clinical setting, and further validates the utility of acquiring a diffusion scan which can quantify both lower order and higher order diffusion models.

Additionally, DTI has shown deficiencies as a biomarker in other previous more acute studies, reporting similar negative TBSS or ROI results in 19 autopedestrian subjects injured within 31 (±20) days, (Wu et al., 2018) 15 college athletes concussed within 30 (±2) days, (Zheng et al., 2014) 23 emergency room subjects within 17 (±7.2) days, (Messé et al., 2011) and 61 level 1 trauma center subjects within 47 (±6.5) days. (Lange et al., 2012) These studies, including ours, may have not have been powered enough to detect a small effect size of FA in mTBI, indicating NODDI to be favorable in detecting differences in studies of smaller sample sizes retaining more controlled patient homogeneity.

Given that previous longitudinal studies have consistently reported progressive WM damage in chronic TBI, (Newcombe et al., 2011, Li et al., 2016) timing after injury is an important consideration when assessing WM alterations. (Yamagata et al., 2020) Therefore the time interval between injury and imaging date (acute versus chronic) may also effect results. Our study was done in a chronic population of subjects at least 3 months after injury, still experiencing post-concussive symptoms. Prior reports of DTI and NODDI measurements in acute TBI have shown both increases in FA (Churchill et al., 2017) and MD (Palacios et al., 2020a, Palacios et al., 2020b) in mTBI less than six months before imaging, in addition to changes in V_ic_ and ODI. These results may differ from those found in our study due to both the mechanism of injury (ex. repetitive sub-concussive hits over long periods of time^16^), and the acute time point of analysis (in some cases, two weeks^13^). Interestingly, although detecting changes in FA and MD in the acute phase of injury (two weeks after injury), longitudinal decreases were observed NODDI values but not in DTI over a 6 month time span, (Palacios et al., 2020a, Palacios et al., 2020b) hence sensitivity of DTI to detect injury may diminish over a longer timespan, where NODDI metrics are still sensitive to pathological alterations. While findings of DTI in TBI have large disparities, the limited number of NODDI studies tend to agree upon the robustness of this technique as a TBI biomarker, despite varying patient cohorts, diffusion pulse sequences, and acquisition protocols. (Wu et al., 2018, Palacios et al., 2018, Gazdzinski et al., 2020, Churchill et al., 2019)

Interestingly, a within-group partial correlation analysis demonstrated that lower V_ic_ and FA values in widespread regions were associated with lower scores in tasks of executive functioning, but not with mood or memory. FA, MD, AD, and RD had a mixture of significant correlations with measures of mood and executive functioning, independent of brain region. In general, while unable to detect significant between-group differences between cTBI and controls, DTI was able to predict neurocognitive outcomes just as well as NODDI metrics, while controlling for age, sex, and time-after-injury. In particular, NODDI shows potential for delineating between group differences, where DTI shows more potential for detecting alterations in mood, self-reported symptoms, and task performance (Table 2**,** Fig. 5). This mixed association finding is supported by previous studies of neurocognitive performances in the chronic stage and DTI metrics, finding varying correlations between diffusion parameters and cognition. (Veeramuthu et al., 2015) This may be attributed to the prolonged functional and neurospychological alterations in subjects with lasting symptoms of TBI, including neuronal network reorganization, (Voets et al., 2006) spurting of axons with smaller calibres, (Jafari et al., 1997) glial scarring, (Stichel and Müller, 1998) and disruptive neurofibrillary tangles. (Stein et al., 2014)

**Table 2:** Significant results of the within-group partial correlation of neuropsychological outcomes with DTI and NODDI measures.

Region		Neuropsychological Measure	Partial Correlation	P-Value (uncorrected)
**DTI**				
	**FA**			
	Corticospinal tract L	A Trails	−0.409	0.013
	Corticospinal tract R	Ability	0.369	0.041
	Cingulum (cingulate gyrus) L	A Trails	−0.369	0.027
	Cingulum (hippocampus) L	A Trails	−0.362	0.027
	Cingulum (hippocampus) R	State Anxiety	0.386	0.029
	Cingulum (hippocampus) R	Sleepiness	0.336	0.045
	Forceps major	A Trails	−0.497	0.002
	Forceps major	B Trails	−0.341	0.042
	Forceps minor	Participation	−0.380	0.035
	Inferior fronto-occipital fasciculus L	A Trails	−0.354	0.034
	Superior longitudinal fasciculus L	A Trails	−0.361	0.030
	Superior longitudinal fasciculus L	B Trails	−0.371	0.026
	Superior longitudinal fasciculus R	B Trails	−0.336	0.045
	Uncinate fasciculus L	Confusion	−0.357	0.048
	Uncinate fasciculus L	Adjustment	−0.402	0.025
	Uncinate fasciculus L	Adaptability	−0.369	0.041
	Uncinate fasciculus L	Sleepiness	−0.347	0.038
	**MD**			
	Corticospinal tract R	Adjustment	−0.426	0.017
	Corticospinal tract R	Adaptability	−0.376	0.037
	Corticospinal tract R	RPQ-13	−0.353	0.032
	Uncinate fasciculus L	Sleepiness	0.336	0.045
	Superior longitudinal fasciculus (temporal part) L	A Trails	0.427	0.009
	Superior longitudinal fasciculus (temporal part) L	B Trails	0.459	0.004
	**AD**			
	Cingulum (hippocampus) L	Confusion	−0.472	0.007
	Cingulum (hippocampus) L	Adjustment	−0.548	0.001
	Cingulum (hippocampus) L	Adaptability	−0.418	0.019
	Inferior fronto-occipital fasciculus L	Participation	0.449	0.011
	Superior longitudinal fasciculus L	Participation	0.406	0.002
	Uncinate fasciculus L	Depression	0.464	0.009
	Superior longitudinal fasciculus (temporal part) L	Participation	0.449	0.011
	Superior longitudinal fasciculus (temporal part) L	A Trails	0.463	0.004
	Superior longitudinal fasciculus (temporal part) L	B Trails	0.573	< 0.001
	**RD**			
	Corticospinal tract R	Confusion	−0.440	0.013
	Corticospinal tract R	Adjustment	−0.545	0.001
	Corticospinal tract R	Participation	−0.440	0.013
	Corticospinal tract R	Adaptability	−0.567	< 0.001
	Forceps major	A Trails	0.427	0.008
	Superior longitudinal fasciculus R	A Trails	0.356	0.033
	Superior longitudinal fasciculus R	Trait Anxiety	−0.372	0.036
	Uncinate fasciculus L	Sleepiness	0.378	0.023
	**ODI**			
	Cingulum (hippocampus) L	Sleepiness	0.352	0.035
	Forceps major	Participation	−0.372	0.039
	Forceps minor	Confusion	0.393	0.029
	Forceps minor	Backward	−0.411	0.010
	Inferior longitudinal fasciculus L	Confusion	0.409	0.022
	Inferior longitudinal fasciculus L	Adaptability	0.382	0.049
	Inferior longitudinal fasciculus L	Sleepiness	0.389	0.019
	Uncinate fasciculus L	Adjustment	0.399	0.014
	Uncinate fasciculus L	Adaptability	0.405	0.012
	**V_ic_**			
	Corticospinal tract L	A Trails	−0.355	0.033
	Corticospinal tract R	A Trails	−0.396	0.017
	Cingulum (cingulate gyrus) L	B Trails	−0.342	0.042
	Forceps major	A Trails	−0.338	0.041
	Inferior longitudinal fasciculus L	A Trails	−0.374	0.025
	Inferior longitudinal fasciculus R	A Trails	−0.503	0.002
	Superior longitudinal fasciculus L	A Trails	−0.610	< 0.001
	Superior longitudinal fasciculus R	A Trails	−0.383	0.021
	Superior longitudinal fasciculus (temporal part) L	Confusion	0.388	0.031
	Superior longitudinal fasciculus (temporal part) L	A Trails	−0.388	0.031

The results in both the between-group and within-group correlation analysis provide evidence that WM axonal density declines within the chronic stages of TBI. Our results showed lower fiber orientation dispersion correlated with worse performances in cognition, consistent with prior studies showing more organized WM in subjects with better intellectual functioning. (Fjell et al., 2011) Due to the exploratory nature of the hypothesis of this study, in addition to the lack of repeated observations on a single subject, multiple comparisons were not performed for the within-group partial correlation analysis. All correlations between diffusion metric and neuropsychological outcome were performed independently and without repeated observations. (Curran-Everett, 2000) These new hypotheses from exploratory findings warrant future studies in larger cohorts using multiple follow-up times to identify the link of white matter changes and time since injury and elucidate the possible recovery of white matter tracts over time. Another limitation of this study is the smaller sample size of controls, as well as the discrepancies in age and sex. The effects of age and sex may confound the interpretation of the between-group differences slightly. In order to mitigate differences in patient population, we have included age and sex as covariates in both the between-group and within-group analysis. However, even when controlling for population source, injury-to-imaging interval, age of injury, sample size, publication date, acquisition parameters, and analysis methods, bidirectional changes in DTI and NODDI parameters have been reported in TBI literature. (Eierud et al., 2014, Dodd et al., 2014) Therefore a more thorough exploration of these variables as they relate to imaging biomarkers and TBI is an area for future research. In addition, the NODDI model uses rigid-stick geometry which may impose constraints with fixed intra-axonal diffusivities and a tortuosity model for extra-axonal diffusion. Future studies modeling axons using diffusion kurtosis imaging, (Fieremans et al., 2011) or q-space measurements (Hosseinbor et al., 2013) utilizing the same HYDI acquisition may add additional information in estimating pathological changes which occur after cTBI, without linking intra- and extra-axonal diffusivities. (Wu et al., 2011)

In summary, these results found that NODDI parameters are more sensitive in detecting between-group differences in the subtle yet complex underlying WM microstructural pathology after cTBI, highlighted by the use of a HYDI scan. Moreover, NODDI may be a more robust clinical biomarker than DTI due to its sensitivity and versatility in detecting pathological WM changes after cTBI in addition to detecting correlations with cognition. NODDI measurements revealed declining neurite density in predominantly posterior WM in a chronic population, which are known to be topologically integral to multiple sensory and cognitive domains including attention and executive functioning. While unable to detect between-group differences, DTI values were significant in predicting neuropsychological outcomes, indicating DTI metrics may be more accurate in the prediction neuropsychological deficits rather than delineation of injured WM pathology. Our results highlight the utility of multi-shell imaging to acquire more sensitive imaging biomarkers for acute and longitudinal diagnosis, and indication of the need for multi-compartment diffusion modeling in addition to the information provided by classical DTI. HYDI is useful imaging tool for clinical translation of DTI biomarkers for the prediction of self-reported symptoms, cognitive performance, and for treatment monitoring, in a broader range of TBI applications.

## Funding

Funding for this study is from a gift from the Marcus Foundation.

## CRediT authorship contribution statement

**Jennifer Muller:** Conceptualization, Methodology, Validation, Formal analysis, Investigation, Resources, Data curation, Writing - review & editing, Visualization, Supervision, Project administration. **Devon Middleton:** Conceptualization, Methodology, Validation, Formal analysis, Investigation, Data curation, Writing - review & editing. **Mahdi Alizadeh:** Methodology, Validation, Formal analysis, Investigation, Data curation, Writing - review & editing. **George Zabrecky:** Resources, Project administration, Funding acquisition. **Nancy Wintering:** Resources, Project administration, Funding acquisition. **Anthony J. Bazzan:** Resources, Project administration, Funding acquisition. **Ji Lang:** Conceptualization, Methodology, Data curation, Writing - review & editing. **Chengyuan Wu:** Investigation, Writing - review & editing, Data curation, Funding acquisition. **Daniel A. Monti:** Conceptualization, Resources, Project administration, Funding acquisition. **Qianhong Wu:** Conceptualization, Investigation, Resources, Data curation, Writing - review & editing, Supervision, Project administration. **Andrew B. Newberg:** Conceptualization, Methodology, Data curation, Writing - review & editing, Supervision, Project administration, Funding acquisition. **Feroze B. Mohamed:** Conceptualization, Methodology, Data curation, Writing - review & editing, Supervision, Project administration, Funding acquisition.

## Declaration of Competing Interest

The authors declare that they have no known competing financial interests or personal relationships that could have appeared to influence the work reported in this paper.

## References

[b0005] Alexander A.L., Wu Y.C., Venkat P.C. (2006). Hybrid diffusion imaging (HYDI). Annu. Int. Conf. IEEE Eng. Med. Biol. - Proc..

[b0010] Arnett J.A., Labovitz S.S. (1995). Effect of physical layout in performance of the Trail Making Test. Psychol. Assess..

[b0015] Asken BM, Dekosky ST, Clugston JR, Jaffee MS, Bauer RM. Diffusion tensor imaging (DTI) findings in adult civilian, military, and sport-related mild traumatic brain injury (mTBI): a systematic critical review. 2018:585-612. doi:10.1007/s11682-017-9708-9. 10.1007/s11682-017-9708-9 28337734

[b0020] Bigler ED. Neuropathology of mild traumatic brain injury: correlation to neurocognitive and neurobehavioral findings.Title. In: Brain Neurotrauma: Molecular, Neuropsychological, and Rehabilitation Aspects. Boca Raton (FL): CRC Press/Taylor & Francis. ; 2015:Chapter 13. 26269912

[b0025] Cercignani M., Bouyagoub S. (2017). Brain microstructure by multi-modal MRI: Is the whole greater than the sum of its parts?. Neuroimage..

[b0030] Churchill N.W., Caverzasi E., Graham S.J., Hutchison M.G., Schweizer T.A. (2017). White matter microstructure in athletes with a history of concussion: Comparing diffusion tensor imaging (DTI) and neurite orientation dispersion and density imaging (NODDI). Hum. Brain Mapp..

[b0035] Churchill N.W., Caverzasi E., Graham S.J., Hutchison M.G., Schweizer T.A. (2019). White matter during concussion recovery: Comparing diffusion tensor imaging (DTI) and neurite orientation dispersion and density imaging (NODDI). Hum. Brain Mapp..

[b0040] Curran-Everett D. (2000). Multiple comparisons: Philosophies and illustrations. Am. J. Physiol. – Regul. Integr. Comp. Physiol..

[b0045] .

[b0050] Dodd A.B., Epstein K., Ling J.M., Mayer A.R. (2014). Diffusion tensor imaging findings in semi-acute mild traumatic brain injury. J. Neurotrauma.

[b0055] Eierud C., Craddock R.C., Fletcher S. (2014). Neuroimaging after mild traumatic brain injury: Review and meta-analysis. NeuroImage Clin..

[b0060] Fieremans E., Jensen J.H., Helpern J.A. (2011). White matter characterization with diffusional kurtosis imaging. Neuroimage.

[b0065] Fjell A.M., Westlye L.T., Amlien I.K., Walhovd K.B. (2011). Reduced white matter integrity is related to cognitive instability. J. Neurosci..

[b0070] Gazdzinski L.M., Mellerup M., Wang T. (2020). White matter changes caused by mild traumatic brain injury in mice evaluated using neurite orientation dispersion and density imaging. J. Neurotrauma.

[b0080] Hosseinbor A.P., Chung M.K., Wu Y., Alexander A.L. (2013). Bessel fourier orientation reconstruction (BFOR): An analytical diffusion propagator reconstruction for hybrid diffusion imaging and computation of q-space indices. Neuroimage.

[b0085] Inglese M., Makani S., Johnson G. (2005). Diffuse axonal injury in mild traumatic brain injury: A diffusion tensor imaging study. J. Neurosurg..

[b0090] Jafari S.S., Maxwell W.L., Neilson M., Graham D.I. (1997). Axonal cytoskeletal changes after non-disruptive axonal injury. J. Neurocytol..

[b0095] Jenkinson M., Beckmann C.F., Behrens T.E.J., Woolrich M.W., Smith S.M. (2012). Review FSL. Neuroimage..

[b0100] Jones D.K., Cercignani M. (2010). Twenty-five pitfalls in the analysis of diffusion MRI data. NMR Biomed..

[b0105] Kraus MF, Susmaras T, Caughlin BP, Walker CJ, Sweeney JA, Little DM. White matter integrity and cognition in chronic traumatic brain injury : a diffusion tensor imaging study. 2007:2508-2519. doi:10.1093/brain/awm216. 10.1093/brain/awm216 17872928

[b0110] Lange R.T., Iverson G.L., Brubacher J.R., Mädler B., Heran M.K. (2012). Diffusion tensor imaging findings are not strongly associated with postconcussional disorder 2 months following mild traumatic brain injury. J. Head Trauma Rehabil..

[b0115] Lerma-Usabiaga G., Mukherjee P., Ren Z., Perry M.L., Wandell B.A. (2018). Replication and generalization in applied neuroimaging. Neuroimage..

[b0120] Levin H.S., Diaz-Arrastia R.R. (2015). Diagnosis, prognosis, and clinical management of mild traumatic brain injury. Lancet Neurol..

[b0125] Li L., Sun G., Liu K. (2016). White matter changes in posttraumatic stress disorder following mild traumatic brain injury: A prospective longitudinal diffusion tensor imaging study. Chin. Med. J. (Engl)..

[b0130] Malec J.F., Brown A.W., Leibson C.L. (2007). The mayo classification system for traumatic brain injury severity. J. Neurotrauma.

[b0135] Maruta J., Palacios E.M., Zimmerman R.D., Ghajar J., Mukherjee P. (2016). Chronic post-concussion neurocognitive deficits. i. relationship with white matter integrity. Front. Hum. Neurosci..

[b0140] Mayer A.R., Ling J., Mannell M.V. (2010). A prospective diffusion tensor imaging study in mild traumatic brain injury. Neurology..

[b0145] Messé A., Caplain S., Paradot G. (2011). Diffusion tensor imaging and white matter lesions at the subacute stage in mild traumatic brain injury with persistent neurobehavioral impairment. Hum. Brain Mapp..

[b0150] Newcombe V., Chatfield D., Outtrim J. (2011). Mapping traumatic axonal injury using diffusion tensor imaging: Correlations with functional outcome. PLoS ONE.

[b0155] Palacios E., Owen J.P., Yuh E.L. (2018). The evolution of white matter microstructural changes after mild traumatic brain injury: A longitudinal DTI and NODDI study. bioRxiv..

[b0160] Palacios E.M., Owen J.P., Yuh E.L. (2020). The evolution of white matter microstructural changes after mild traumatic brain injury: A longitudinal DTI and NODDI study. Sci. Adv..

[b0165] Palacios E., Owen J.P., Yuh E.L. (2020). The evolution of white matter microstructural changes after mild traumatic brain injury: A longitudinal DTI and NODDI study. Sci. Adv..

[b0170] Radhakrishnan R., Garakani A., Gross L.S. (2016). Neuropsychiatric aspects of concussion. Lancet Psych..

[b0175] Stein T.D., Alvarez V.E., McKee A.C. (2014). Chronic traumatic encephalopathy: A spectrum of neuropathological changes following repetitive brain trauma in athletes and military personnel. Alzheimer’s Res Ther..

[b0180] Stichel C.C., Müller H.W. (1998). The CNS lesion scar: New vistas on an old regeneration barrier. Cell Tissue Res..

[b0185] Veeramuthu V., Narayanan V., Kuo T.L. (2015). Diffusion tensor imaging parameters in mild traumatic brain injury and its correlation with early neuropsychological impairment: A longitudinal study. J. Neurotrauma.

[b0190] Voets N.L., Adcock J.E., Flitney D.E. (2006). Distinct right frontal lobe activation in language processing following left hemisphere injury. Brain.

[b0195] Wallace E.J., Mathias J.L., Ward L. (2018). Diffusion tensor imaging changes following mild, moderate and severe adult traumatic brain injury: a meta-analysis. Brain Imaging Behav..

[b0200] Wu Y, Harezlak J, Flashman LA, Mcallister TW. Hybrid Diffusion Imaging in Mild Traumatic Brain Injury. 2018;2390:2377-2390. doi:10.1089/neu.2017.5566. 10.1089/neu.2017.5566 PMC6196746 29786463

[b0205] Wu Y.C., Field A.S., Whalen P.J., Alexander A.L. (2011). Age- and gender-related changes in the normal human brain using hybrid diffusion imaging (HYDI). Neuroimage.

[b0210] Xiao M., Ge H., Khundrakpam B.S. (2016). Attention performance measured by attention network test is correlated with global and regional efficiency of structural brain networks. *Front*. Behav. Neurosci..

[b0215] Yamagata B., Ueda R., Tasato K. (2020). Widespread White Matter Aberrations Are Associated with Phonemic Verbal Fluency Impairment in Chronic Traumatic Brain Injury. J. Neurotrauma.

[b0220] Zhang H., Schneider T., Wheeler-kingshott C.A., Alexander D.C. (2012). NeuroImage NODDI: Practical in vivo neurite orientation dispersion and density imaging of the human brain. Neuroimage..

[b0225] Zheng Z., Shemmassian S., Wijekoon C., Kim W., Bookheimer S.Y., Pouratian N. (2014). DTI correlates of distinct cognitive impairments in Parkinson’s disease. Hum. Brain Mapp..

[b0230] Zhou Z., Li X., Kleiven S. (2020). Biomechanics of Periventricular Injury. J. Neurotrauma.

